# Modification of the Intrastromal Ring Position according to Postimplant Visual and Refractive Failure: Report of Two Cases

**DOI:** 10.1155/2019/7151849

**Published:** 2019-06-04

**Authors:** Luis Izquierdo, Josefina A. Mejías Smith, Jose R. Lievano, Ramón Sarquis, Maria A. Henriquez

**Affiliations:** ^1^Head of Department of Cornea and Anterior Segment, Instituto de Ojos Oftalmosalud, Lima, Peru; ^2^Research Department, Instituto de Ojos Oftalmosalud, Lima, Peru; ^3^Head of Research Department, Instituto de Ojos Oftalmosalud, Av. Javier Prado Este 1142, San Isidro, Lima, Peru

## Abstract

**Objective:**

The following report presents the adaptation of an existing technique of intrastromal corneal ring (ICRS) implantation enabling repositioning of the ring position postoperatively to manage a refractive failure in two patients with keratoconus.

**Methods:**

In two cases, KeraRing and Ferrara nomograms had suggested different ring positions. To manage with the differences between the two nomograms, a longer corneal tunnel was created followed by the classic intervention to move the ring through the initial intrastromal corneal tunnel according to the topographic values. Once the first ring position has failed, the ring segment was repositioned along the longer corneal tunnel according to the postoperative outcome.

**Results:**

Significant improvement in both cases was observed in the best corrected visual acuity (BCVA), uncorrected visual acuity (UCVA), and root mean square (RMS) measured with Scheimpflug imaging (Pentacam; Oculus GmBH, Wetzlar, Germany). The participants were followed for one year.

**Conclusion:**

In patients with keratoconus which exhibit significant differences between KeraRing and Ferrara nomograms, a longer tunnel should be created to enable repositioning of the ring postoperatively if necessary, to avoid extracting the ring or changing it.

## 1. Introduction

Keratoconus is a noninflammatory, bilateral asymmetric eye disease in which the cornea progressively thins and forms a cone-like bulge. The disease typically involves the central two-thirds of the cornea; the apex of the cone usually centered just below the visual axis. The disease results in mild to severe impairment of visual function due to the development of progressive myopia and irregular astigmatism.

Keratoconus management depends on the progression and stage of the disease. In mild cases, it can be managed with crosslinking, eyeglasses, phakic lenses, photorefractive keratotomy (PRK), or contact lenses. In moderate cases, when the disease progression has stopped, the visual axis is clear and the patient's corrected-vision is not sufficient to perform his daily life activities and intracorneal ring segments (ICRS) can be used. Deep anterior lamellar keratoplasty (DALK) or penetrating keratoplasty (PK) can be used in severe cases when the visual axis is compromised by corneal scarring, best corrected visual acuity is 20/100 or worse, and the pachymetry is less than 300 um [1, 2].

The purpose of the implant is to reshape the corneal surface and improve tolerance to contact lenses and their related symptoms such as itching and eye dryness. Moreover, it can also delay or even prevent corneal transplantation.

ICRS are usually indicated for moderate keratoconus and high myopia cases, improving the best corrected visual acuity (BCVA), decreasing the keratometry values, remodeling the corneal topography, and delaying corneal keratoplasty [3–5]. Complications related to ICRS include extrusion, keratitis, corneal melting, perforations, and refractive failure [6]. Refractive failure is a term used to describe a poor postoperative outcome defined by a minimum loss of one line in uncorrected visual acuity (UCVA) or BCVA, an increase of two diopters of spherical equivalent, and an increase of 1 mm in the root mean square (RMS) [7].

To define both the meridian, the depth, and the implant area of the intrastromal ring segment, one must rely on the nomograms supplied by the manufactures of each ring [8, 9].

We performed implantations using both Ferrara and KeraRing nomograms. According to the KeraRing nomogram [8], the first step is to select the so-called reference meridian. If the best correction visual acuity (BCVA) is equal or greater than 0.5 logMAR, the refractive meridian (perpendicular to the refractive axis in negative) is selected; if the BCVA is less than 0.5 logMAR, the meridian of the coma is selected (perpendicular to the coma axis). The second step is to determine the type of corneal asymmetry considering the chosen meridian reference and the axial keratometric (curvature) map, followed by determining the slope area of one side of the selected reference meridian. According to this relation, four types of positions are described according to the percentage of unevenness area associated with the reference meridian.

For Ferrara nomogram the strategy is based on asphericity [9]. If the asphericity is less than -0.25 a 160° corneal segment is implanted on the topographically flatter axis; if the asphericity is between -0.25 and -1.25, two segments are implanted; if the asphericity is greater than -1.25, a 210° segment is implanted in the 90° axis; this classification continues dividing the cones into central or paracentral.

The following report presents the adaptation of an existing technique of intrastromal corneal ring (ICRS) implantation enabling repositioning of the ring position postoperatively to manage a refractive failure in two patients with keratoconus.

## 2. Case**** 1

**Figure 1:**
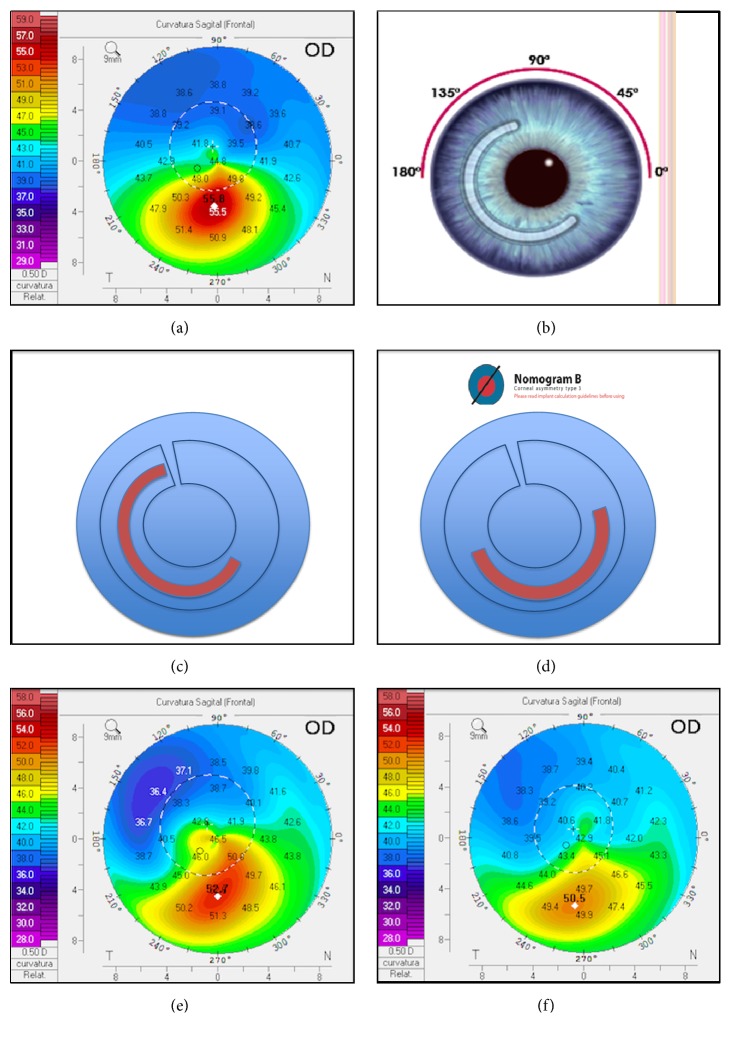
(a) Topography pretreatment. (b) Ferrara nomogram. (c) First intrastromal ring position. (d) KeraRing nomogram position. (e) One-month postimplantation ring position. (f) One-year postmodification ring position.

A 27-year-old male with keratoconus presented at the Cornea and Anterior Segment Department with blurred vision that was difficult to correct with eyeglasses. In his right eye, his UCVA was 20/100 and his manifest refraction was -0.75-1.25 x 50° with BCVA of 20/30. In his left eye, his UCVA was 20/25 with -0.25-0.50 x 75° and BCVA of 20/20 (Figure 1(a)). ICRS was implanted in his right eye and crosslinking in his left eye.

The ICRS (KeraRing, Mediphacos, Belo Horizonte, Brazil) used in this study was a 160° segment ring made of polymethyl methacrylate (PMMA) implanted in the corneal tunnel (Figure 1(c)). Both ICRS nomograms, Ferrara and KeraRing, indicated different ring position (Figures 1(b)-1(d)), so a temporal 160°/200-micron ring was utilized. A femtosecond laser (Z6; Ziemer Ophthalmic Systems AG, Port, Switzerland) was used to make an incision at 114° (the steepest meridian), K1 42.3 D, and K2 47.4 D, and a 371um deep corneal tunnel was created (corresponding to 75% of the corneal thickness of 495 um at the thinnest point of the tunnel path), with a 355-degree length only 20 degrees more than the normal size of the ICRS. This longer channel allowed repositioning of the ring if necessary.

The thickness and degree of arc of the ICRS were selected and their location was planned according to the cone location on axial topography measured via Scheimpflug imaging (Pentacam; Oculus GmBH, Wetzlar, Germany), with a 5.5 mm diameter depending on the nomogram results.

Postoperative treatment included 0.3% topical tobramycin with 0.1% dexamethasone (Tobradex; Alcon, Fort Worth, TX, USA) four times a day for 2 weeks; the latter was then tapered over 4 weeks. Preservative-free artificial tear substitute (Lagricel Ofteno; Sophia, Guadalajara, Mexico) was used four times a day for 2 weeks.

Two months later the patient's UCVA was 20/100, his BCVA was 20/30, and manifest refraction was +1.50-1.75 x 10°, indicating a refractive failure. However, his RMS improved from 14.750 um to 10.346 um (Figure 1(e)).

According to the postoperative keratometric Scheimpflug map, the ring was moved 50 degrees counterclockwise according to the topographic results (Figure 1(d)). One-month after repositioning, the patient's UCVA was 20/25, his BCVA was 20/20, his manifest refraction was -0.50 x 90°, and his RMS had decreased to 5.392 um.

At a one-year follow-up, his right eye was UCVA 20/30-2, his manifest refraction was +1.00-2.00 x 90 degrees with BCVA of 20/20, and his RMS was 5.605 um (Figure 1(f)).

## 3. Case**** 2

**Figure 2:**
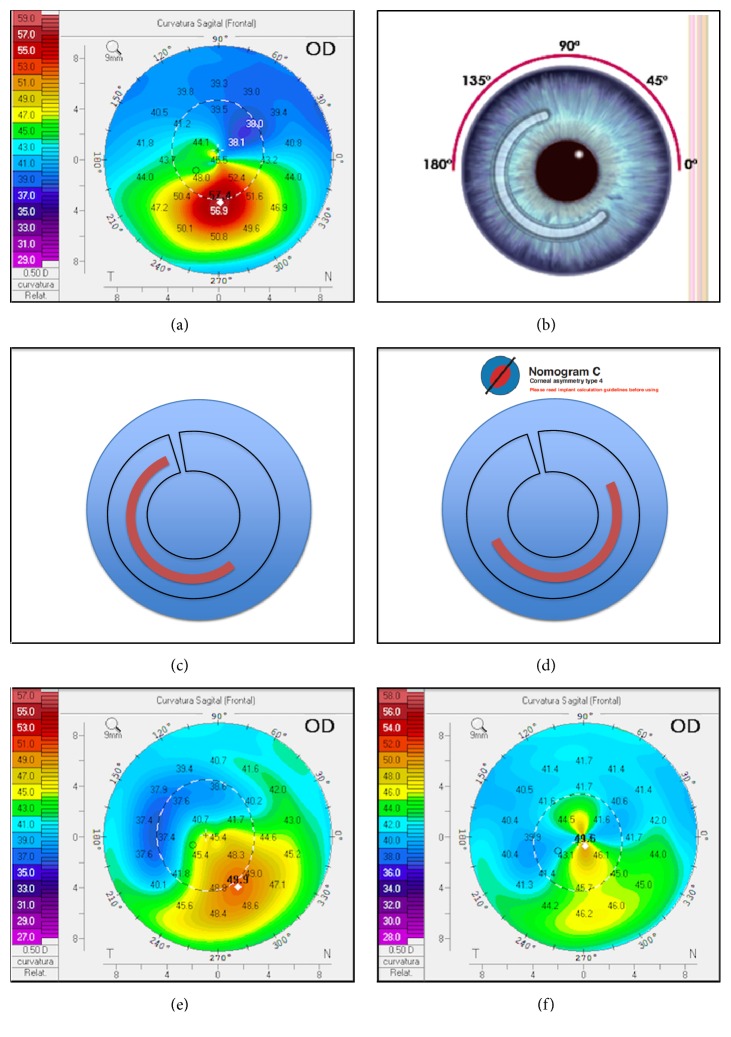
(a) Topography pretreatment. (b) Ferrara nomogram. (c) First intrastromal ring position. (d) KeraRing nomogram position. (e) One-month postimplantation ring position. (f) One-year postmodification ring position.

A 26-year-old male presented at the Cornea and Anterior Segment Department with low vision in the right eye, which presented UCVA of 20/100 BCVA 20/30 and manifest refraction of -0.75-3.5 x 50°. His left eye had UCVA of 20/25, BCVA of 20/20, and manifest refraction of -0.25-0.50 x 75°. Keratoconus was diagnosed in both eyes; femto ICRS was indicated for the right eye and follow-up for the left eye (Figure 2(a)).

The two nomograms proposed, KeraRing and Ferrara, indicated different ring positions (Figures 2(b)-2(d)), so a temporal 160°/150-micron ring (Figure 2(c)) was utilized. A femtosecond laser (Z6; Ziemer Ophthalmic Systems AG, Port, Switzerland) was used to make an incision at 99.7° (the steepest meridian by topography), K1 42.5 D, and K2 46.1 D. Due to a pachymetry of 512 microns at the tunnel zone, a 371 um deep corneal tunnel (corresponding to 75% of the corneal thickness) was created with a 355-degree tunnel if repositioning was necessary.

Postoperative treatment included 0.3% topical tobramycin with 0.1% dexamethasone (Tobradex; Alcon, Fort Worth, TX, USA) four times a day for 2 weeks; the latter was then tapered over 4 weeks. Preservative-free artificial tear substitute (Lagricel Ofteno; Sophia, Guadalajara, Mexico) was used four times a day for 2 weeks.

Two months later, the patient's UCVA was 20/150, his BCVA was 20/40, and his manifest refraction was -1.00-2.00 x 50°, indicating a loss of one line of vision (Figure 2(e)). Additionally, his RMS decreased from 13.970 um to 11.327 um. The patient's outcome was not as planned, and his visual acuity was worse after the ICRS implantation. Therefore, the ring was moved 45 degrees counterclockwise in the tunnel according to the keratometric Scheimpflug map (Figure 2(d)).

One month after repositioning, the patient's UCVA improved to 20/40, his BCVA to 20/20, and his manifest refraction to +0.50-2.00 x 90°. His RMS decreased from 11.327 um to 8.275um.

At a one-year follow-up, his right eye was UCVA 20/40-2, his manifest refraction was +0.50-2.00 x 90 with BCVA of 20/25+2, and his RMS was 7.569 um (Figure 2(f)).

## 4. Discussion

As indicated by the clinical data from both cases, the tunnel adaptation for the ICRS implantation has the potential to correct myopia, astigmatism, and corneal irregularity.

This adaptation provides opportunities to manage keratoconus to avoid refractive outcomes and ring explantation. The procedure is simple, has no additional cost, and can be used for manual or femtosecond tunnels.

Torquetti et al. evaluated 37 keratoconus eyes implanted with intrastromal corneal ring segments and described requirements for reintervention following overcorrection due to excessive flattening of the cornea with unsatisfactory results in all of the cases and limited improvement after the first procedure [10].

Coskunseven et al. [11] studied 50 eyes of patients with keratoconus using KeraRing ICRS and reported segment migration to the incision site in three eyes. To avoid melting, they repositioned the migrated segment away from the incision site with successful outcomes in all of the eyes. Heikal et al. reported no complications or need for ring repositioning in a 6-month follow-up of 20 patients [12].

Pokroy et al. [13] reported that 10% of keratoconic eyes with implanted ring segments needed adjustments (repositioning, exchange, or explantation) due to surgically induced astigmatism. The adjustment procedure was technically simple with no intraoperative complications.

Chan et al. [14] evaluated 3 case series of patients with previous insertion of 2 intrastromal corneal ring segments who underwent surgical removal and repositioning of the segments due to unsatisfactory visual and topographic outcomes. Although the results were good, the removal of the intrastromal ring and the creation of a new tunnel could have been avoided.

We believe when managing patients with significant differences between nomograms, a longer tunnel should be created to enable repositioning of the ring postoperatively if necessary. This technique may reduce the need in additional operations and improve the patient's refractive outcomes.
